# Brain Magnetic Resonance Imaging of Children With Molybdenum Cofactor Deficiency

**DOI:** 10.1002/jimd.70079

**Published:** 2025-08-31

**Authors:** B. C. Schwahn, R. Sinha, J. A. M. Wright, J. Pavaine

**Affiliations:** ^1^ Willink Metabolic Unit, Manchester Centre for Genomic Medicine St Mary's Hospital, Manchester University NHS Foundation Trust, Health Innovation Manchester Manchester UK; ^2^ Division of Evolution and Genomic Sciences Faculty of Biology, Medicine and Health, University of Manchester Manchester UK; ^3^ Imaging Department Royal Preston Hospital, Lancashire Teaching Hospitals NHS Foundation Trust Preston UK; ^4^ School of Medicine/Mental Health and Clinical Neuroscience, Precision Imaging Hub, Medical School Queen's Medical Centre, University of Nottingham Nottingham UK; ^5^ Imaging Department University Hospitals Plymouth NHS Trust Plymouth UK

**Keywords:** brain imaging, cyclic pyranopterin monophosphate, excitotoxicity, hypoxic ischemic encephalopathy, molybdenum cofactor deficiency, MRI

## Abstract

Molybdenum cofactor deficiency (MoCD) is a rare differential diagnosis of neonatal hypoxic ischemic encephalopathy (HIE) with considerable variation in presentation and treatment outcomes. The temporospatial evolution of brain MRI appearances has not been well described. We systematically evaluated 35 MRI brain scans of 13 patients with neonatal MoCD (7 type A, 6 type B) to characterize brain abnormalities arising from exposure to toxicity related to sulfite accumulation and to evaluate changes in response to cPMP treatment in 6 children with MoCD type A. All cases showed evidence of chronic toxicity with developmental disruption. We identified a disease‐specific pattern of acute and chronic brain injury, distinct from HIE. White matter edema, as the earliest sign of sulfite‐related toxicity, indicates a reversible disease stage. The presence of restricted diffusion in the context of MoCD signifies irreversible brain injury and a poor neurological prognosis, irrespective of subsequent biochemical correction upon cPMP treatment. This is the largest neuroimaging study of children with MoCD and the first longitudinal study to examine MR imaging changes in MoCD type A under cPMP substitution. Neuroimaging can identify diagnostic and prognostic features with relevance for treatment decisions and for the evaluation of the effectiveness of treatment attempts.

## 1 Introduction

Molybdenum cofactor deficiency (MoCD, OMIM ID # 252150) is a genetic disorder with a birth prevalence of less than 1 in 100,000 [1]. While MoCD constitutes a rare differential diagnosis to neonatal hypoxic–ischemic encephalopathy (HIE), a correct diagnosis is crucial for appropriate patient management. Children with MoCD can already be encephalopathic at birth but typically appear well and manifest after a short interval with acute encephalopathy and seizures, with subsequent evolution of neurological sequelae, including cortical visual impairment, cognitive disability, and dystonic and spastic tetraplegic cerebral palsy [2, 3]. Some newborns with severe MoCD present subacutely, within weeks after birth, show cystic encephalomalacia in brain imaging despite a lack of evidence of an acute brain insult, and go on to develop severe cerebral palsy. Rarely, older children present with atypical and attenuated symptoms of variable severity [2, 3, 4, 5].

MoCD causes impaired sulfite oxidase activity with raised sulfite concentrations. Animal and in vitro studies have demonstrated sulfite‐mediated direct mitochondrial impairment [6, 7, 8, 9] and excitotoxic injury through formation of S‐sulfocysteine (SSC) which is a potent agonist at ionotropic glutamate receptors [10, 11, 12, 13]. A postnatal surge in sulfite and SSC is likely responsible for the peracute onset of symptoms that reflect neuronal and glial injury.

The universally poor prognosis of severe MoCD type A changed when treatment with cyclic pyranopterin monophosphate (cPMP) became available [14, 15, 16] which rapidly restores the function of molybdenum cofactor dependent enzymes and corrects the biochemical disturbance [17]. A small number of children with severe MoCD‐A have been treated with cPMP [18] and those treated pre‐symptomatically have escaped significant neurological disease [15, 16, 19]. Other patients treated after the onset of severe acute encephalopathy have developed typical disease sequelae [14, 16].

Neuroimaging features of MoCD and isolated sulfite oxidase deficiency have been reported, and the chronic disease stage has been well documented [20, 21, 22, 23, 24]. A recent meta‐analysis has indicated the possibility of multiple, sequential acute events during pregnancy and after birth [25]. A systematic evaluation of the temporal and spatial evolution of brain MRI changes in the acute and subacute phase of the disease and of long‐term outcomes after biochemical correction using cPMP in MoCD type A has, however, not been performed.

## 2 Patients and Methods

### 2.1 Patients

We systematically evaluated 35 brain MRI scans and one brain CT scan of 13 patients born at term with confirmed severe MoCD and typical, early‐onset presentation (7 MoCD‐A and 6 MoCD‐B), who underwent brain imaging as part of their clinical care by the authors. Sixteen MRI scans were obtained from 10 patients without specific treatment, including 4 patients with MoCD‐A prior to cPMP treatment (at age 3, 4, 6, and 42 days) and 6 patients with MoCD‐B (at age 1 to 131 days). Another 19 MRI scans (at age 5 to 2264 days) were obtained from 6 patients with MoCD‐A who received treatment with cPMP on a named‐patient basis with parental consent. Table 1 provides an overview of the timing of brain scans in relation to cPMP treatment, and Table S1 shows pertinent clinical characteristics of the patient population. Clinical aspects of some of the included patients have been previously published: Patients A and G [16], Patient B [19], Patients D, E, I [26], Patients K and M [27].

**TABLE 1:** Overview of brain 35 MRI scans in 13 patients with MoCD in relation to symptom onset and cPMP treatment.

Patient ID	MoCD type	Gestational age at birth in weeks	Brain scans at postnatal age in days									
A	A	39	A1 (3)				A2 (93)		A3 (414)	A4 (814)		
B	A	38.5	B1 (4)	B2 (14)		B3 (44)		B4 (135)		B5 (780)	B6 (1639)	B7 (2264)
C	B	39		C1 (11)		C2 (40)						
D	A	39	D1 (6)	D2 (10)	D3 (21)							
E	A	40	E1 (5)		E2 (20)					E3 (1030)		
F	B	39	F1 (3)									
G	A	41			G1 (18)		G2 (91)		G3 (395)			
H	A	40				H1 (42)						
I	A	39	I1 (6)		I2 (24)	I3 (48)						
J	B	40			J1 (23)			J2 (131)				
K	B	39	K1 (1)	K2 (10)		K3 (44)						
L	B	37.5	L1 (6)									
M	B	38.3	M1 (1)					M2 (261)		M3 (604)		

*Note:* Scan E3 is an additional brain CT scan. Shaded background denotes ongoing cPMP substitution at the time of imaging. Numbers in parentheses give the age at imaging in days of life.

### 2.2 Stratification of Disease Stages

We stratified MR images according to time after onset of first disease symptoms. Adapted from the classification of HIE, the acute phase was defined as 0–7 days after onset of acute encephalopathy, and the chronic phase as 28 days and longer after onset, with weekly strata in between.

### 2.3 MR Imaging and Evaluation

Brain MRI scans were performed according to standard neonatal and pediatric protocols. Standard locations were assessed by two pediatric neuroradiologists for structural abnormalities, signal alterations for vasogenic and cytotoxic edema, brain necrosis (liquefaction), cystic transformation, hemorrhage, and brain atrophy.

Vasogenic edema was evidenced by increased T2 and reduced T1 signal, and increased signal in a diffusion weighted image (DWI) without a decreased Apparent Diffusion Coefficient (ADC) signal.

Cytotoxic edema with impending tissue breakdown was evidenced by increased DWI signal and decreased ADC signal. Normal ADC reference intervals are 850–1300 × 10^−6^ mm^2^/s for deep grey structures, 1200–1800 for periventricular white matter (PWM), and 1000–1400 for cerebral cortex [28]. ADC values were not calibrated using a standard phantom, due to variable scanning conditions for individual patients and acquisition of images over the course of over 10 years.

## 3 Results

### 3.1 Congenital Structural Brain Abnormalities Indicate Fetal Developmental Disruption of the Posterior Fossa, Brainstem, and Cerebellum

**FIGURE 1:**
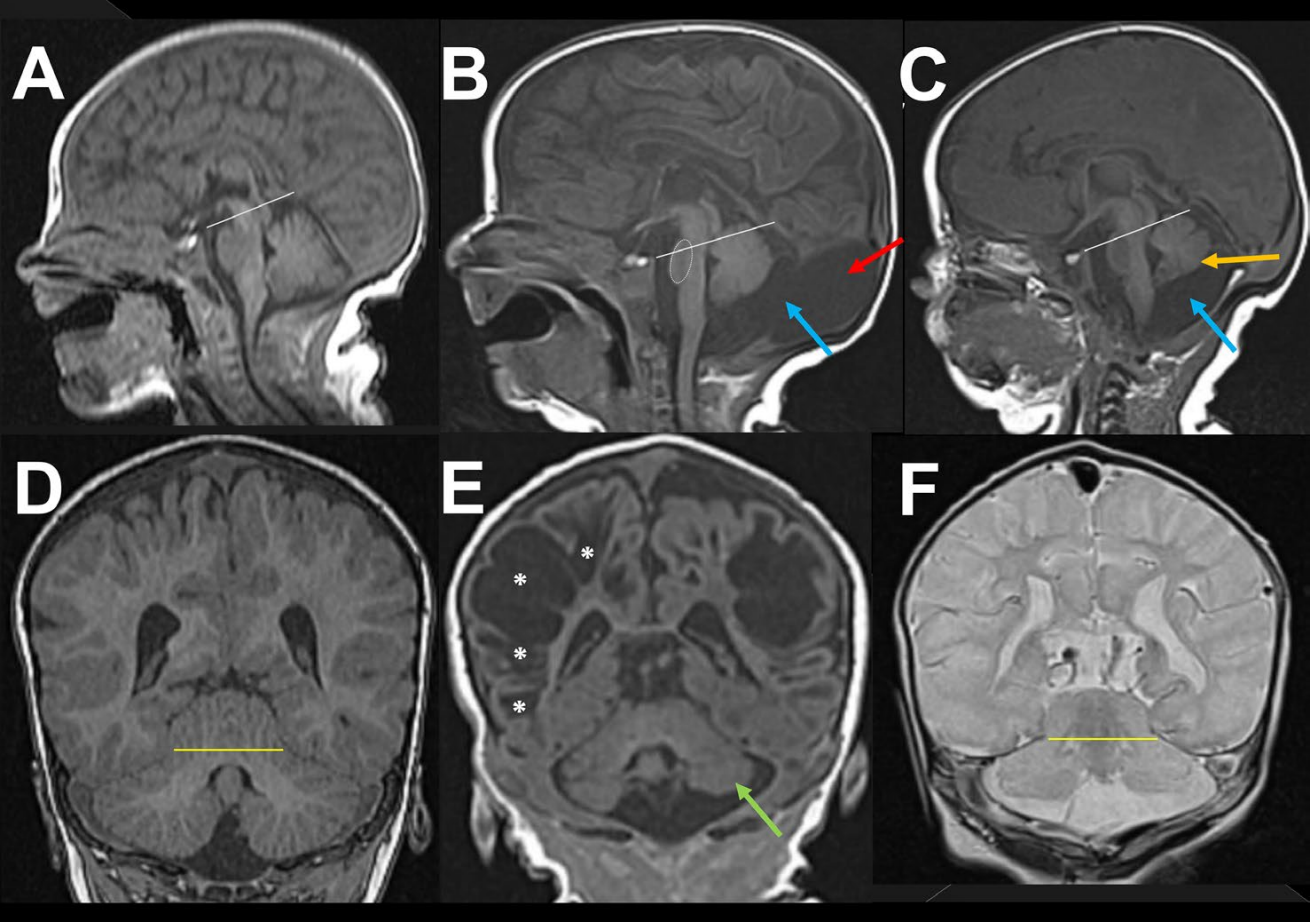
Characteristic findings in the posterior fossa of neonates with molybdenum cofactor deficiency. (A) Sagittal midline T1WI shows normal brainstem and cerebellum in control neonate at 18 DoL. White line—incisural line from dorsum sellae to Vein of Galen/straight sinus junction; note incisural line traverses midbrain in the mid‐point. (B) Sagittal T1WI shows mega cisterna magna (blue arrow). Note posterior dysplasia of the tentorium cerebelli with cystic dilatation/expansion of MCM extending supratentorially through posterior tentorial hiatus (red arrow). Note pontine hypotrophy with small pons and flattened pontine protuberance (dotted circle). Note shift upward of the midbrain and superior pons across incisural line (white line). (C) Sagittal T1WI shows mega cisterna magna without abnormality of the tentorium cerebelli (blue arrow). Note midbrain shift upward across incisural line (white line). Note inferior vermis position projecting above the obex indicative of vermian hypoplasia (orange arrow). (D) Coronal T1WI depicts superior cerebellar towering across tentorial incisura (yellow line). (E) Coronal T1WI shows mildly smaller left cerebellar hemisphere (green arrow) compared to the right side, contralateral to the right cerebral hemisphere with more extensive cystic encephalomalacia (asterisks), reflecting crossed cerebellar diaschisis. (F) Coronal T2WI shows small for age cerebellum. Note superior cerebellar towering across tentorial incisura (yellow line).

The first available brain MRI scan (*N* = 13, median age at scan 6 days, range 1–42) was assessed for structural abnormalities. None showed evidence of brain malformation. We, however, identified a disruption of posterior fossa development in all 13 patients (Figure 1). A mega cisterna magna, characterized by a depth of the cisterna magna above 10 mm in the presence of an intact cerebellum [29], was found in 6 of 13 patients (A, B, D, I, K, M) to a variable extent (Figure 1, Panels B, C). Three (A, D, K) of those had an additional posterior dysplasia of the tentorium with cystic expansion of the cisterna magna supratentorially across a posterior tentorial hiatus (Figure 1, Panel B). Small‐for‐age cerebellar hemispheres were found in all 13 patients (Table S2) and 9 had variable cerebellar vermian hypotrophy (C, D, E, F, I, J, K, L, M). A smaller inferior than superior vermian height was observed in 3 of these (E, I, M) (Figure 1, Panel C).

All patients had evidence of cerebellar crossed diaschisis (Figure 1, Panel E). In most patients, the brainstem and cerebellum appeared to be cranially displaced, with a shift of the midbrain upward, and towering of the cerebellum across the tentorial incisura (Figure 1, Panels D, F). Normally, the incisural line (from the tip of the dorsum sellae turcicae to the attachment of the vein of Galen on the straight sinus) crosses the midpart of the midbrain (Figure 1, Panel A); [30]. However, the entire midbrain was located above the incisural line in 11 of 13 patients (except patients H, J) and variable portions of the upper pons extended above the incisural line in 7 (A, B, C, D, F, I, M) out of these 11 patients (Figure 1, Panels B, C). Variable pontine hypotrophy with flattening of the pontine protuberance and a prominent prepontine cistern was observed in all patients (Figure 1, Panels B, C).

### 3.2 Vasogenic Edema Is Present in the Early Stage of Sulfite Toxicity and Can Persist for at Least 6 Weeks

Signs of vasogenic edema were consistently found in deep white matter (DWM) on the first available images of all 13 patients (age range 1–42 days). In untreated patients, vasogenic edema persisted at least until the age of 40 (C2), 42 (H1), and 44 days (K3). Signs of vasogenic edema were absent at the age of 131 days in patient J (J2). There were no suitable brain MRI scans available of older patients.

### 3.3 Widespread and Persisting Cytotoxic Edema Characterizes the Second Stage of Sulfite Toxicity in MoCD

Signs of cytotoxic edema were found in the first scan in 11 of 13 patients, at age 1 to 42 days.

Patient A had no restricted diffusion in any part of the brain; in Patient B, we found only mild reduced diffusivity in the cerebral cortex (B1), which was not present anymore at age 14 days (B2).

**FIGURE 2:**
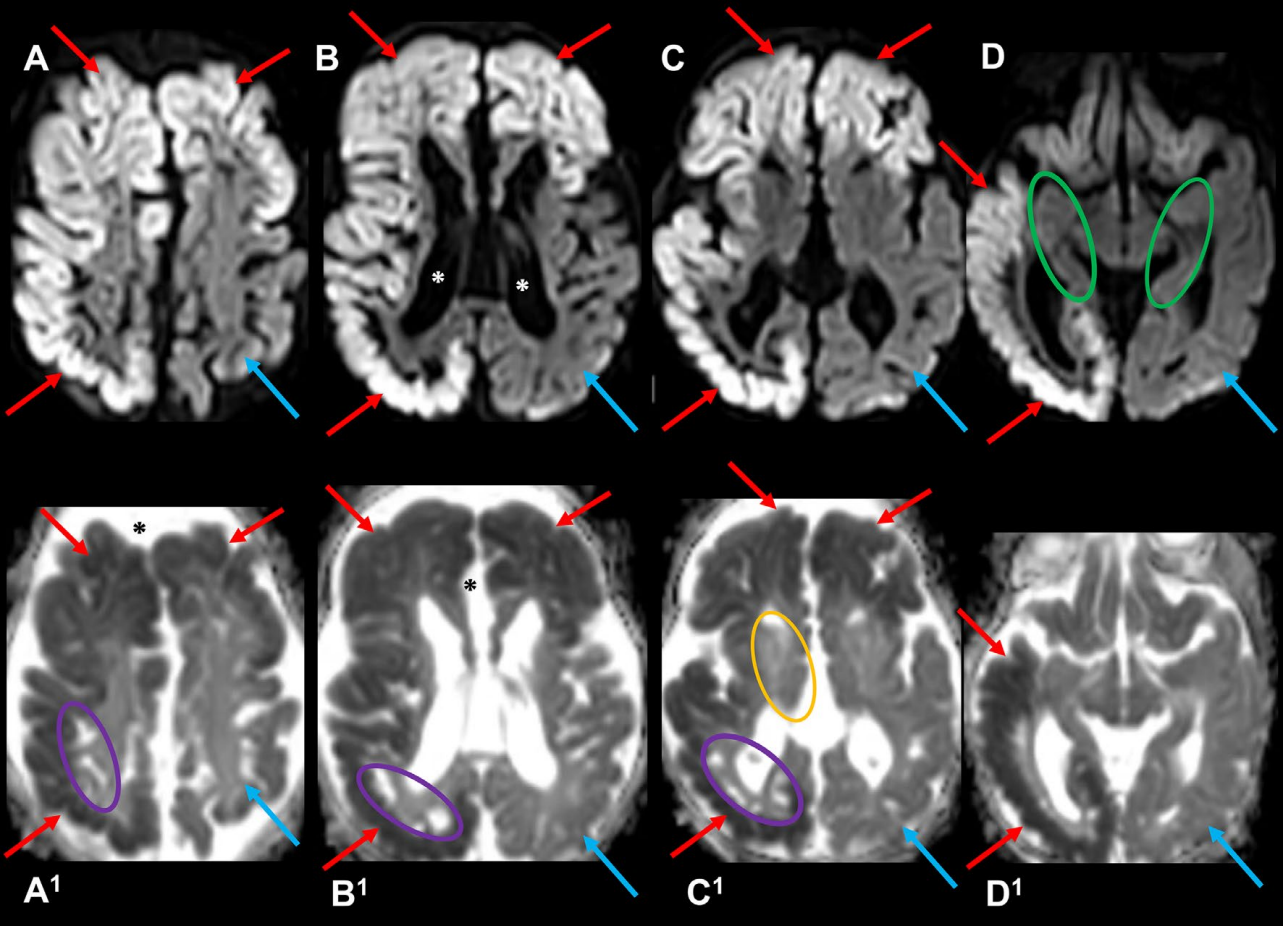
Asymmetric restricted diffusion superimposed on established brain atrophy and cystic encephalomalacia in Patient H. Upper row (A–D): axial DWI at age 42 days. Bottom row (A^1^–D^1^): Corresponding axial ADC map. High DWI/low ADC signal denotes cortical and subcortical WM cytotoxic edema in the right cerebral hemisphere and left frontal lobe (red arrows). No restricted diffusion is seen in the left‐sided parietal, temporal, and occipital lobes (blue arrows). Mesial temporal lobes are preserved (green ovals). Dilated lateral ventricles (white asterisks) and enlarged pericerebral CSF subarachnoid spaces (black asterisks) indicate brain atrophy. Note basal ganglia and thalamic atrophy (orange oval). Established cystic transformation is demonstrated in the parietal, temporal, and occipital subcortical WM (violet ovals).

The other 11 patients showed widespread involvement of cerebral hemispheres, with earlier involvement of the posterior lobes and later affection of frontal lobes and sparing of mesial temporal lobes in all patients. The presence of severe diffusion restriction correlated with concomitant clinical signs of pronounced acute encephalopathy (Table S1). Diffusion restriction was typically symmetric but was strikingly asymmetric in two patients superimposed on already established cystic encephalomalacia and atrophy evidencing sequential acute hits (H1, L1). In Patient L (L1 age 6) asymmetric cortico‐subcortical restricted diffusion was unilateral, and bilateral in Patient H (H1 age 42) (Figure 2).

**FIGURE 3:**
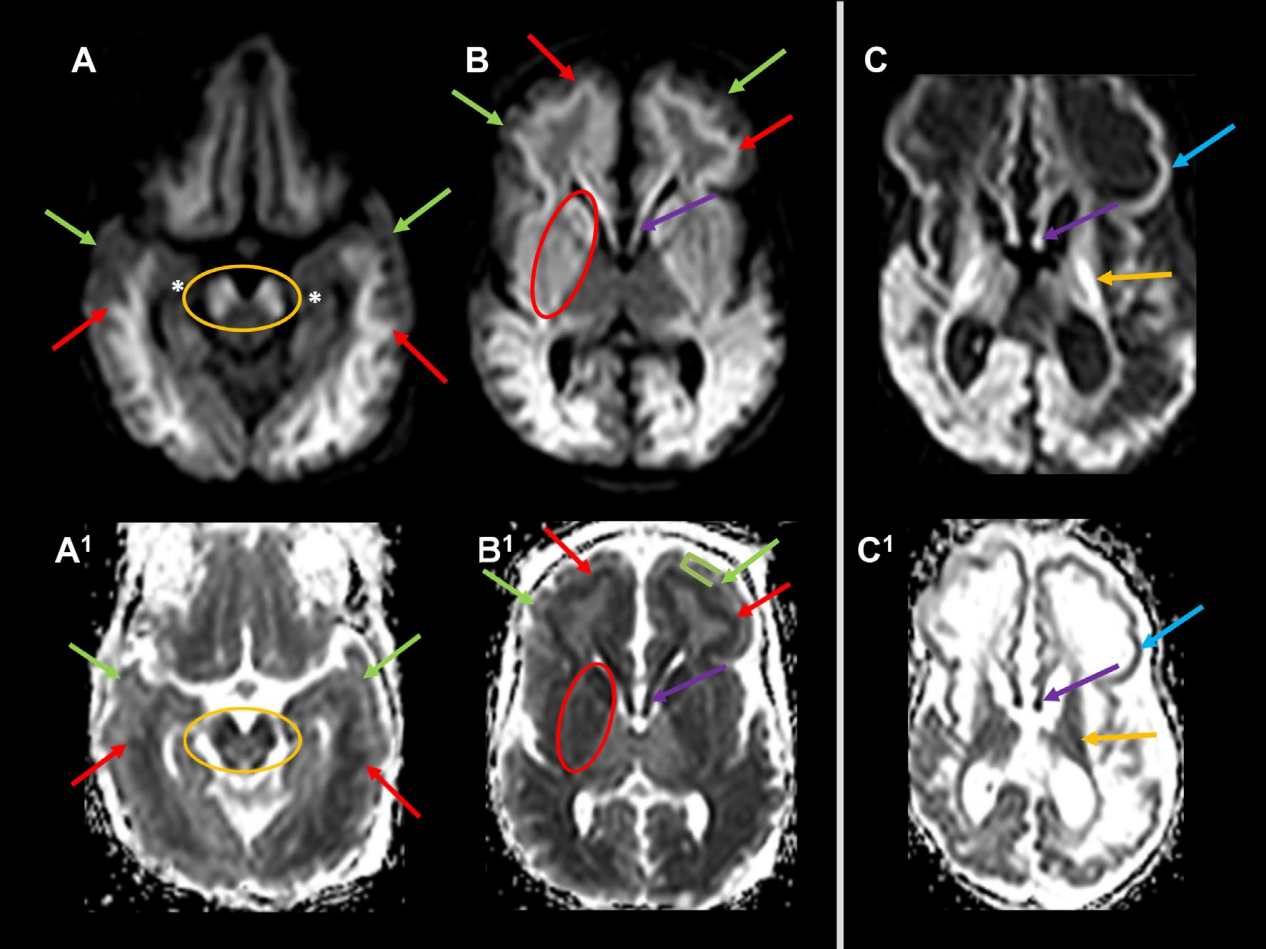
Juxtacortical WM acute injury pattern in Patient C. A–A^1^, B–B^1^: At age 11 days; C–C^1^: At age 40 days. Upper row (A–C): Axial DWI; Bottom row (A^1^–C^1^): Corresponding axial ADC map. A–A^1^: Axial DWI/ADC map at midbrain/temporo‐occipital lobes level. B–B^1^, C–C^1^: Axial DWI/ADC map at basal ganglia level. A–A^1^, B–B^1^: High DWI/low ADC signal evenly underlying entire cerebral cortex indicates juxtacortical WM cytotoxic edema at age 11 days (red arrows). The overlying frontal and temporal cortex is swollen with no restricted diffusion (green arrows, bracket). Mesial temporal lobes are preserved (asterisks). Note restricted diffusion in cerebral peduncles (orange oval), basal ganglia (red oval). C–C^1^: High DWI/low ADC signal in the cerebral cortex indicates cortical restricted diffusion/cytotoxic edema at age 5 weeks (blue arrows). Note interval cerebral atrophy with SWM cystic transformation. Restricted diffusion in the posterior limbs of internal capsules progressed (orange arrows); restricted diffusion in fornix persists (violet arrows).

Extensive restricted diffusion was evident in the cerebral cortex and subcortical white matter (SWM) in 11 patients. Patients C (C1 age 11), D (D1 age 6), E (E1 age 5) and K (K1 age 1) initially showed relative preservation of the anterior frontal cortex with signs of vasogenic edema in comparison with the underlying SWM with signs of cytotoxic edema. In addition, the anterior temporal cortex was relatively spared in C1 and K1. Patient C shows the most striking difference between cortical and SWM involvement. Their initial scan (C1 age 11 days) showed avid restricted diffusion in fronto‐temporal SWM; however, the overlying cortex was swollen with no associated restricted diffusion. While the fronto‐temporal cortex appeared relatively spared initially, follow‐up imaging (C2 age 40 days) revealed cortical restricted diffusion (Figure 3). Follow‐up imaging of Patients C, D, E, and K showed cortical thinning and evidence of cortical laminar necrosis implying interim cytotoxic edema.

Patients showed evidence of cytotoxic edema in basal ganglia, along WM tracts in corpus callosum (CC), posterior limbs of internal capsules (PLIC) and the brainstem. The extent of diffusion restriction in grey matter was increasing over the course of the first week after birth and persisted for up to 2–3 weeks in deep grey matter and 4–6 weeks in cortical grey matter, suggesting an ongoing disease process in untreated patients (Table 2 and Table S3). Cortical restricted diffusion was present in untreated patients at age 40 (C2), 42 (H1), and 44 days (K3), and residual frontal cortical restricted diffusion was seen in treated Patient I at age 48 days (I3). The severity and extent of restricted diffusion in cerebral WM and BG appeared less in follow‐up scans of untreated Patients C (C2 age 40) and K (K3 age 44) and of treated Patient E (E2 age 20) due to transformation into cystic and necrotic areas.

**TABLE 2:** Spatial prevalence of diffusion restriction in brain MRI, corresponding to time after onset of clinical signs of disease in 11 patients with molybdenum cofactor deficiency and onset of symptoms on day 1 of life. Patient L already showed signs of established cystic encephalomalacia at age 6d, prenatal onset of acute encephalopathy was assumed and the scan assigned to disease stage 3. Data are presented as percentage of affected patients per disease stage. Green 0% – 25% affected, Yellow: 26% – 50% affected, Orange: 51% – 75% affected. Red: 76% – 100% affected.

		Cerebral hemispheres				Deep grey structures				Brainstem		
						Basal ganglia						
**Disease stage (N MRI)**		Cortical grey matter (CGM)	Subcortical white matter (SWM)	Corpus callosum (CC)	Posterior Limb of Internal capsule (PLIC)	Caudate Nucleus (CN)	Putamen (Put)	Globus pallidus (GP)	Thalamus (Thal)	Midbrain (MB)	Pons (PO)	Medulla oblongata (MO)
**1**	within 7 days of onset (6)	100	83	83	83	100	83	83	83	83	83	83
**2**	7 – 13 days after onset (3)	100	100	100	100	100	100	100	100	100	100	100
**3**	14 – 20 days after onset (3)	100	67	67	67	33	33	33	67	67	67	67
**4**	21 – 27 days after onset (2)	100	0	0	100	50	50	50	0	50	100	0
**5**	28 days and more after onset (4)	100	50	0	50	0	0	0	25	0	0	0

### 3.4 Cortical Laminar Necrosis, Brain Liquefaction and Cystic Transformation Follow in Areas With Severe Diffusion Restriction

Patient A and B never had restricted diffusion nor displayed cystic changes during their follow‐up.

No imaging signs of necrosis or cystic transformation were found in the initial scans of patients C, D, E, F, I, K, and M (age 1–11 days). Patients F and L were not re‐scanned after the first scan at age 3.5 and 6 days, respectively. Evidence of cortical laminar necrosis, WM liquefaction, and cyst formation was later found in all other 9 patients. Patients C, D, E, I, K, and M, with evidence of cytotoxic edema in their first scan (age 1–11 days) had developed necrotic or cystic changes seen in their next scan at age 20 (E2), 21 (D3), 24 (I2), 40 (C2), 44 (K3) and 261 (M2) days. The MRI appearance suggested onset of acute severe brain injury at or shortly after birth, in keeping with their clinical presentation. Patient K (K2, age 10 days), Patient I (I2, age 24 days; I3, age 48 days), and Patient E (E2, age 20 days) also showed necrosis in the globi pallidi and putamina.

Patients G, H, J, and L already showed imaging signs of cystic encephalomalacia when their first brain MRI scans were performed at ages 18, 42, 23, and 6 days, respectively. Patient G had a clear history of postnatal onset of acute severe encephalopathy; however, the other three patients had no such history, suggesting a late intrauterine acute event.

**FIGURE 4:**
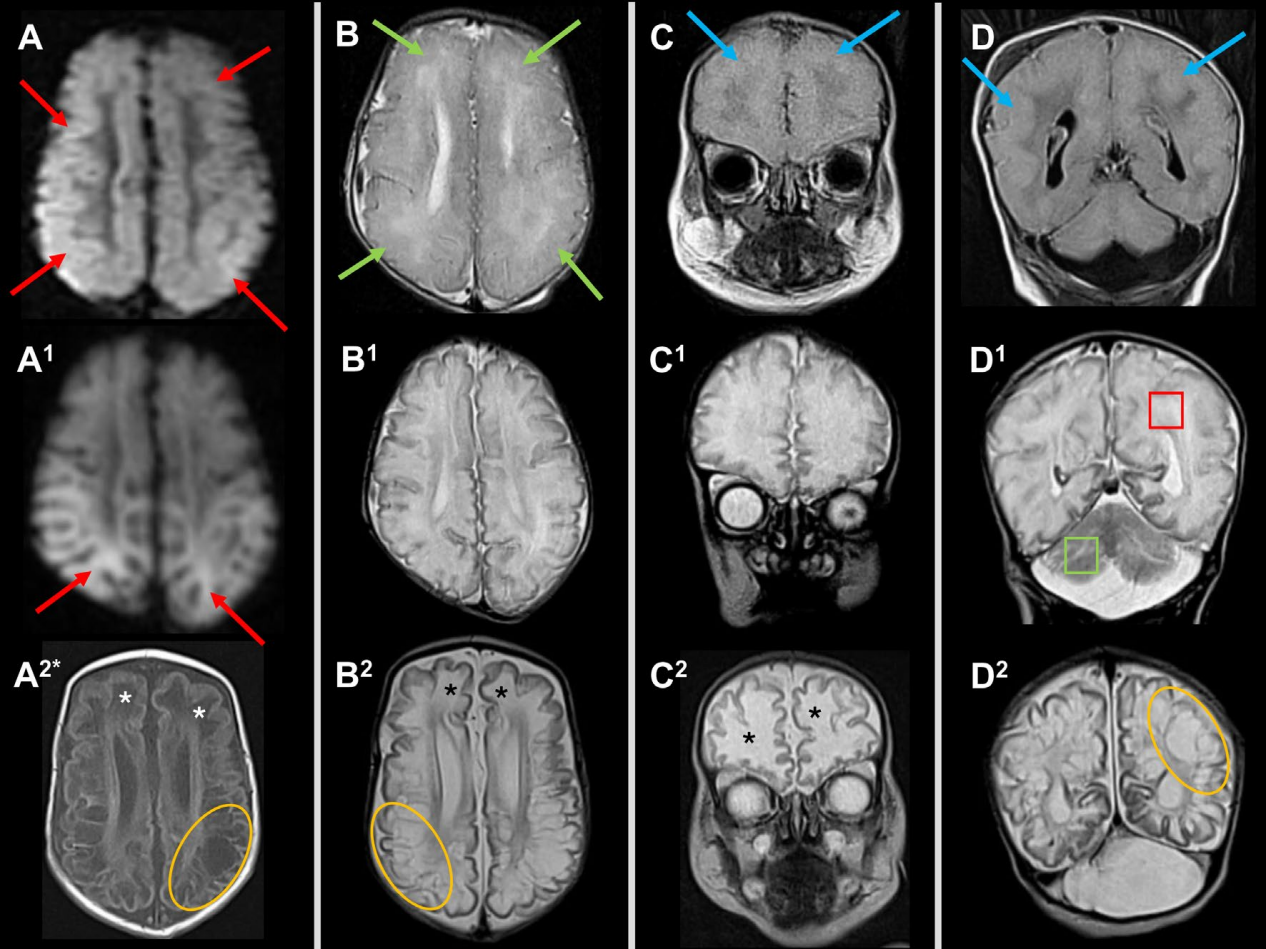
Transition from cytotoxic edema through liquefaction to cystic transformation in an infant with MoCD (Patient D). Upper row (A–D): At age 6 days; Middle row (A1–D1): At age 10 days; Bottom row (A2*–D2): At age 21 days. 1st column: (A–A1): Axial DWI, (A2*): Axial T1WI. DWI sequence was not obtained at age 21 days. 2nd column: (B–B2): Axial T2WI of the frontoparietal lobes. 3rd column: (C–C2): (C) Coronal T2 FLAIR; (C1–C2) Coronal T2WI through the frontal lobes. 4th column: (D–D2): (D) Coronal T2 FLAIR; (D1–D2) Coronal T2WI through the parietal, posterior temporal, and occipital lobes. A–A1: High DWI signal/restricted diffusion in the frontoparietal cortex and subcortical white matter reflect cytotoxic edema at age 6 and 10 days (red arrows). Note restricted diffusion persists with posterior gradient, more striking in the posterior frontal and parietal lobes at age 10 days (A1). B–D: Mildly reduced cortico‐subcortical T2 signal (green arrows)/increased T2 FLAIR signal (blue arrows) compared to deep WM in the swollen cerebral hemispheres with loss of cortical ribbon and sulcal effacement at age 6 days. Note increased water content in deep WM reflecting vasogenic edema. B1–D1: Reduced degree of brain swelling with visualization of previously effaced cerebral sulci. Note high T2 signal in the swollen cerebral white matter (red quadrant) compared to lower T2 signal intensity in the cerebellum (green quadrant); (D1). B2–D2: High uniform T2 signal in the anterior frontal lobes compatible with CSF signal along the sulci represents liquefaction (black asterisks)—a pre‐stage before cystic transformation (B2, C2). Simultaneously, cystic transformation occurred earlier in the posterior frontal, parietal, temporal, and occipital lobes (orange ovals) (B2, D2). Note interval cerebral atrophy with prominent CSF spaces along the sulci and dilated lateral ventricles at age 21 days. A2*: Low T1 signal in the anterior frontal lobes reflects liquefaction (white asterisks), while cystic transformation occurred with posterior gradient in the parietal and posterior frontal lobes (orange oval).

Parieto‐occipital SWM underwent cystic transformation earlier, while bifrontal SWM cytotoxic edema progressed to a transitional stage of liquefaction and showed cystic transformation somewhat later (D3, E2, I2). The transition from cytotoxic edema, through a transitional phase of liquefaction, to cystic transformation in Patient D is illustrated in Figure 4.

### 3.5 Intracerebral Hemorrhage and Subdural Bleed or Effusions Are Secondary Phenomena

Four patients showed birth‐related subdural, intraventricular hemorrhage, or white matter microhemorrhages in their first MRI scan (E1, H1, I1, K1).

Four patients developed subdural effusions or bleeds during the subacute phase secondary to interval cerebral atrophy. We found bilateral frontal subdural effusions at a later age in Patient A (A2), Patient I (I2 and I3) and Patient J (J2). Patient G showed bilateral frontotemporal subdural bleeds (G2).

### 3.6 Evolution of Brain Atrophy in the Chronic Stage

MRI changes indicating brain atrophy were present in all patients with brain imaging in the chronic phase apart from patients A and B. Atrophy was already seen in scans performed at 3–6 weeks of age. Atrophy affected cortical and deep grey matter and white matter and was observed in areas that had previously shown restricted diffusion. A thin corpus callosum (CC) was found in all patients from the first imaging. In some patients, the CC was initially interpreted as normal size (D1, E1, I1). However, on close inspection, in these patients, the CC volume was increased due to cytotoxic edema.

### 3.7 Impact of cPMP Treatment

**FIGURE 5:**
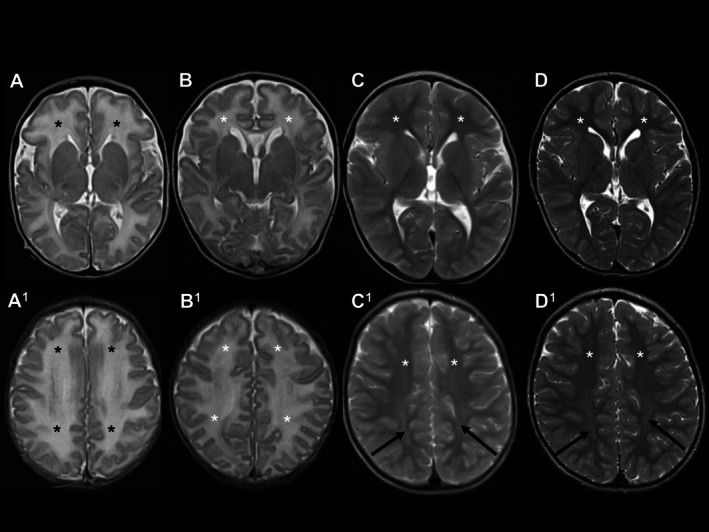
Resolution of vasogenic edema after initiation of treatment with cPMP in Patient B. Upper row (A–D): Axial T2WI at the gangliocapsular level. Bottom row (A1–D1): Axial T2WI at the centrum semiovale. 1st column (A–A1): At age 4 days, 2nd column (B–B1): At age 2 weeks, 3rd column (C–C1): At age 3 years, 4th column (D–D1): At age 6 years. A–A1: Increased T2 signal in the white matter indicates vasogenic edema (black asterisks). B–B1: Reduced T2 signal in the white matter indicates resolved vasogenic edema (white asterisks). C–C1, D–D1: Gradual T2 signal reduction in the white matter indicates normally progressed myelination (white asterisks). C1, D1: On the background of age‐appropriate myelination, increased T2 signal in the posterior frontal and parietal white matter indicates a lack of myelin deposition bilaterally (white arrows).

Patient B had signs of WM vasogenic edema present prior to treatment at age 4 days (B1) which had resolved 9 days after starting cPMP treatment (B2, age 14 days) (Figure 5, second column). We observed a mild degree of brain swelling initially with subsequent minimal volume loss but no evidence of brain atrophy in all imaging follow‐up. Increased T2 signal in SWM and DWM persisted until the age of 2 years, and was not accompanied by restricted diffusion (Figure 5, third and fourth columns).

Patient A was started on cPMP treatment at a more advanced but still oligosymptomatic clinical disease stage. He had signs of vasogenic edema but no diffusion restriction at age 3 days (A1). The first follow‐up scan showed a resolution of the vasogenic edema accompanied by bi‐frontal volume loss with shallow subdural effusions (A2, 93 days), which resolved on further follow‐up, and brain growth appeared intact at age 814 days (A4). Patient A had an abnormal appearance of the peritrigonal white matter associated with focal volume loss over time (A3 and A4).

Patient A and B showed a gradual increase in CC bulk with progression of WM myelination on cPMP treatment. However, at last follow‐up at 2 years 2 months of age (A4) and 6 years 2 months of age (B7), respectively, the posterior CC remained thin for age. Both showed normal brain growth, had normal motor development without movement disorder. However, they were affected with delayed speech development and mild learning difficulties.

**FIGURE 6:**
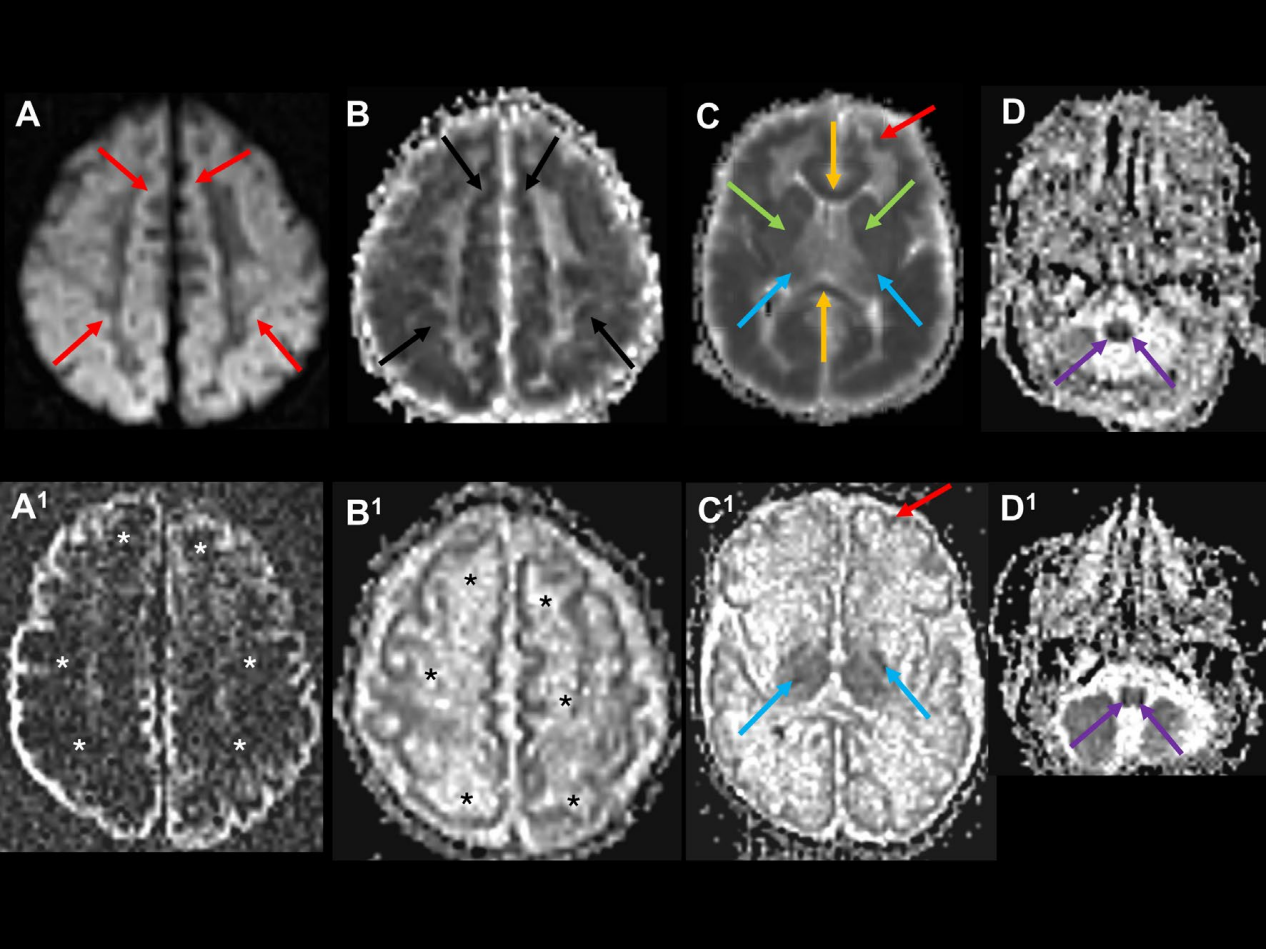
Persistence of cytotoxic edema in a patient with MoCD‐A treated with cPMP after onset of brain injury (Patient E). Upper row (A–D): At age 5 days; Bottom row (A1–D1): At age 20 days. A–B, A1–B1: DWI/ADC map at the centrum semiovale level. C–C1: ADC map at gangliocapsular level. D–D1: ADC map at the medulla oblongata level. A–B: Bilateral high DWI signal (red arrows) with corresponding low ADC (white arrows) demonstrates restricted diffusion in the frontoparietal cortex and subcortical white matter, indicating cytotoxic edema. Note, anterior frontal subcortical white matter is more affected than cortex on ADC map. A1–B1: Bilateral increased DWI/reduced ADC in the frontoparietal cortex indicates residual cytotoxic edema. Note resolved restricted diffusion in the subcortical white matter (asterisks). C: Bilateral low ADC signal/restricted diffusion indicates cytotoxic edema in the cortex and subcortical white matter (red arrow), callosal genu and splenium (orange arrows), basal ganglia (green arrows) and thalamo‐capsular region (blue arrows). C1: Bilateral low ADC signal indicates residual restricted diffusion in cortex (red arrow) and thalamo‐capsular regions (blue arrows). Note resolved restricted diffusion in subcortical white matter, corpus callosum, and basal ganglia. D: Bilateral low ADC signal/restricted diffusion indicates cytotoxic edema in the medulla oblongata (violet arrows). D1: Bilateral low ADC signal in the medulla oblongata is more discernible, indicative of ongoing cytotoxic edema in the medulla oblongata 16 days after treatment initiation (violet arrows).

The long‐term outcome was different for Patients D, E, G, and I, who were treated with cPMP after the onset of severe encephalopathy and seizures. All four patients developed severe spastic and dystonic cerebral palsy, like untreated patients. Signs of vasogenic edema were still present 13, 14, and 16 days after treatment start in some (G1, D3, E2) and had disappeared at age 91 days in one patient (G2) (Figure S1, Panel B). Signs of vasogenic edema were absent at age 48 days, 23 days after treatment discontinuation in Patient I (I3). Patient D was treated at age 7.5 days, 7 days after acute onset, and presented with cystic transformation, atrophy of both cerebral hemispheres, BG, and Thal, during follow‐up at the age of 21 days (D3). Patient E was started on treatment at age 3.5 days, 2 days after the onset of symptoms. Despite treatment at an early age, there was already evidence of widespread cytotoxic edema in the first MRI (E1, age 5 days) (Figure 6, Panels A–D) and a subsequent scan (E2, age 20 days) showed residual restricted diffusion in the cerebral cortex and thalamo‐capsular regions and signs of ongoing cytotoxic edema in the medulla oblongata (Figure 6, Panels A^1^–D^1^). This patient had a CT head scan performed at the age of 2 years 9 months (E3) which revealed pronounced cerebral atrophy with ventriculomegaly and thin CC, cystic encephalomalacia, and small hyperdense BG and thalami.

Patient G was started on treatment at age 5 days, 4 days after the onset of encephalopathy, and the initial brain scan (G1, age 18 days) already showed cystic encephalomalacia and atrophy of CC and BG. Atrophy of both cerebral hemispheres and thalami (G2, age 91 days) progressed on ongoing treatment. Surprisingly, Patient G showed a temporary recurrence of vasogenic edema in DWM bilaterally under treatment, at the age of 395 days (G3), but no restricted diffusion. There was no clinical correlate of acute encephalopathy, although the patient had experienced an increased frequency of seizures and more dystonia in the preceding weeks. On this occasion, the single voxel MR spectroscopy from a frontoparietal DWM showed an increased lactate peak that had been absent in previous imaging (Figure S1). Recurrent vasogenic edema could relate to secondary effects of increased seizure activity. Patient I was started on cPMP at age 4 days, 3.5 days after symptom onset [26]. The first scan at age 6 days (I1) revealed widespread diffusion restriction, and at age 24 days (I2) cystic encephalomalacia and generalized brain atrophy [5]. At the age of 48 days (I3) we observed further parenchymal volume loss with shrinkage of the encephalomalacic cysts [5].

## 4 Discussion

Newborns with MoCD have a normal brain size [16], typically appear well at the time of birth, and rapidly succumb to progressive epileptic encephalopathy with subsequent cerebral palsy [4]. This clinical pattern suggests an acute toxic event after birth, and it has often been suggested that the brain MRI appearance resembles that of profound perinatal hypoxic‐ischemic brain injury (HIE) [22, 23, 24, 31, 32, 33, 34, 35]. However, on closer inspection, the injury pattern in MoCD differs from HIE and suggests chronic as well as acute toxic injury.

### 4.1 Intrauterine Developmental Disruption Is Seen in All Cases

Fetal brain imaging has revealed isolated cerebral cysts and abnormalities of the posterior fossa in selected cases [2, 21, 22, 36, 37, 38, 39, 40, 41]. Increased cerebral white matter T2 signal during late stages of pregnancy [42] and early postnatal cystic encephalomalacia suggesting brain injury prior to birth [23, 43] have been reported.

In our series, we found strong evidence of intrauterine chronic toxicity in all 13 patients, consisting of a cranially displaced midbrain, smaller cerebellum than expected for age, with evidence of crossed diaschisis, and enlarged peri‐cerebellar CSF spaces in the posterior fossa. Crossed cerebellar hypotrophy or diaschisis [44] indicates a pathological process affecting intrauterine and early neonatal cerebellar development due to a compromise of cortico‐ponto‐cerebellar fibers, leading to more pronounced hypotrophy of the cerebellar hemisphere contralateral to the earlier affected cerebral hemisphere. This is typically found after prenatal injury during a particular vulnerable window from 24 to 30 weeks of gestation [45]. We saw predominant hypotrophy of cerebellar hemispheres with relative preservation of vermis. This likely relates to asynchronous patterns of differentiation [46], for example, earlier maturation of Purkinje cells in the vermis than in hemispheres [45]. Differences in the temporospatial expression of NMDA and AMPA/kainate receptors can also explain variable vulnerability to SSC‐mediated excitotoxic effects at different stages of development in different brain areas. A high prevalence of prenatal brain injury with multiple acute events was hypothesized from a recent meta‐analysis of published case reports of MoCD, based on asymmetries of acute changes, although without longitudinal data [25].

### 4.2 Implications for Mechanisms of Toxicity

Neuroimaging in MoCD suggests a disease process affecting both neurons and glial cells. Cortical laminar necrosis and restricted diffusion in deep grey matter reflect neuronal loss. This has been observed in brain injury related to neuronal energetic failure; for example, due to hypoxia, cyanide poisoning, ischemia, hypoglycemia, or increased energy demands due to status epilepticus [47, 48, 49]. In MoCD, it likely reflects stress exerted on neurons by both increased seizure activity and impaired mitochondrial function on the background of SSC‐mediated excitotoxicity [12] and direct toxic effects of sulfite on the mitochondrial respiratory chain [7, 9]. Early and substantial volume loss of SWM with cystic transformation linked to direct loss of glial cells and atrophy of DWM tracts have been interpreted as Wallerian degeneration. Like neurons, astrocytes, microglia, and oligodendrocytes are expressing glutamate receptors and transporters and are vulnerable to disturbances in glutamate signaling. NMDA and AMPA/kainate receptors are mediating excitotoxic injury in pre‐myelinating oligodendrocytes [50, 51] which can cause white matter injury [52, 53]. SSC, as an excitatory amino acid, acts on both NMDA and AMPA/kainate receptors. Even small amounts of accumulating SSC could have a detrimental effect because SSC has no access to the high‐affinity glutamate re‐uptake systems [10].

We postulate that both the direct effects of sulfite and the low‐grade excitotoxic effects of SSC are responsible for chronic intrauterine toxicity. In contrast, the postnatal surge of sulfite and SSC is likely responsible for the acute severe encephalopathy seen in most cases. Restricted diffusion was accompanied by a volume increase sustained at 10 days of life in Patient D (Figure 4, Panels A^1^–D^1^) which indicates persistent cell swelling and oncotic necrosis rather than apoptotic changes in pathological terms [54, 55, 56].

Indirect evidence suggests a late intrauterine onset of acute toxic injury in some patients, including signs at first scan of an advanced stage of brain disease without adequate postnatal clinical correlate (Patients H, J and L) and asymmetries of diffusion restriction (H1, L1). The first scan of Patient J (J1, age 23 days) showed signs of vasogenic edema on the background of established cystic encephalomalacia but no SWM diffusion restriction, evidence of cortical laminar necrosis predominantly in peri‐rolandic regions, and basal ganglia atrophy with clefts in lentiform nuclei. The posterior falx cerebri was deviated to the right, and the left‐sided occipital and posterior temporal lobes were larger than the right‐sided; cystic changes were more extensive in the right cerebral hemisphere than in the left, and the right thalamus was smaller. The left cerebellar hemisphere was smaller than the right. All these findings suggest that the right cerebral hemisphere was affected earlier than the left and to a greater extent, and point to late antenatal injury.

A recent meta‐analysis of case reports [25] suggested that acute brain injury in molybdenum cofactor deficiency can occur repeatedly in one individual at different times of life, including during intrauterine development, and highlighted that injuries can be asymmetric. Our results confirm these observations; although we report a higher proportion of intrauterine developmental disruption, particularly affecting the posterior fossa in all patients. In our series, acute changes superimposed on already established cystic encephalomalacia and atrophy were present in four patients [G, H, J and L], implying sequential acute events. We have found imaging evidence suggestive of prenatal onset of the first acute injury in three patients [H, J and L], and of sequential postnatal acute injury events in one patient [G] accompanied by a postnatal clinical history of prolonged acute severe encephalopathy. Sequential events will likely be masked if a patient suffers from global excitotoxic injury early after birth and prior intrauterine brain imaging was not performed.

### 4.3 Differentiation of MoCD From Hypoxic Ischemic Encephalopathy (HIE)

**FIGURE 7:**
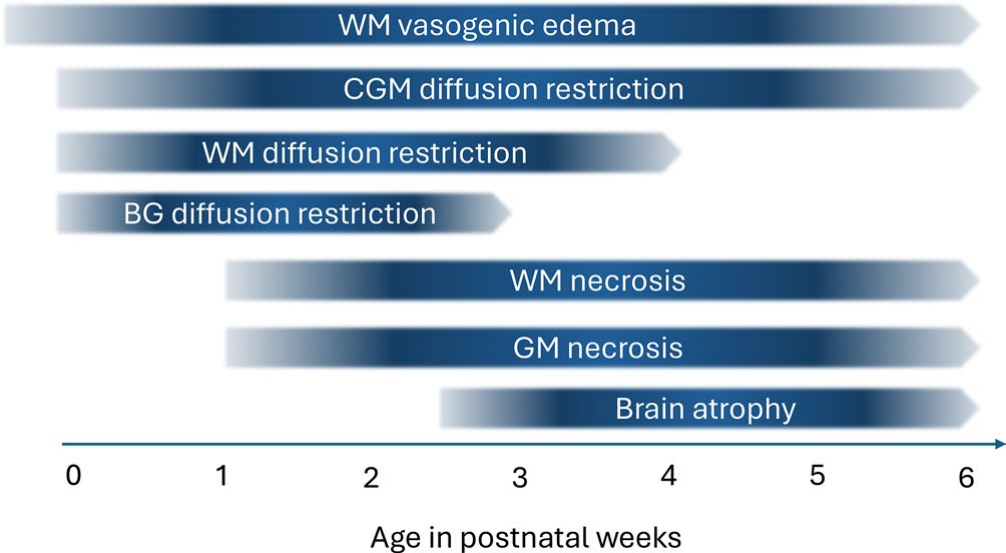
Timeline of observed brain MRI changes in newborns with MoCD. BG: Basal ganglia; CGM: Cortical gray matter; GM: Gray matter; WM: White matter.

We have established a timeline of events (Figure 7) and identified temporospatial differences in the brain MRI appearance of MoCD compared with HIE both in the acute and subacute stages.

#### 4.3.1 Vasogenic Edema Persists for Weeks

All patients in our study had signs of white matter vasogenic edema in their initial scans, even those in a pre‐ or oligosymptomatic phase (A1, B1). Vasogenic edema reflects the earliest sign of sulfite intoxication. Signs of vasogenic edema persisted for at least 6 weeks in untreated patients, which is much longer than expected after a hypoxic ischemic event and signifies an ongoing toxic process. While we only report a small number of observations, this finding is highly unusual and was consistently seen in all patients in our cohort.

#### 4.3.2 Diffusion Restriction in MoCD Indicates Irreversible Cell Death

Widespread diffusion restriction was seen as early as 1 day after birth, with earliest involvement of the basal ganglia, SWM, the posterior cerebral cortex, and progression with a postero‐anterior gradient under sparing of the cerebellum. This pattern distinguishes MoCD from typical global HIE. Brain areas with restricted diffusion invariably progressed to necrosis in all our patients. Diffusion restriction in the context of MoCD therefore represents cytotoxic edema rather than intramyelinic edema, which occurs in Maple Syrup Urine Disease and other toxic encephalopathies (e.g., vigabatrin‐related) and is usually reversible [57].

Diffusion restriction has not been reported prenatally in MoCD. We would predict this to occur, based on the observation of very early cystic transformation in some neonates with a sulfite intoxication disorder [23, 43], also seen in our Patient L at age 6 days. Fetal MRI would be sensitive enough to detect diffusion restriction but has rarely been performed in affected fetuses.

#### 4.3.3 Diffusion Restriction in MoCD Persists Longer and Follows a Different Pattern Compared with HIE

Whereas diffusion restriction in perinatal HIE typically persists for 5 days, with pseudonormalization occurring at 6–8 days [58], in our series, diffusion restriction persisted much longer, for at least 4 weeks in SWM and 6 weeks in CGM after the onset of severe encephalopathy and seizures. Of note, neonates with perinatal HIE undergoing therapeutic hypothermia show prolonged restricted diffusion up to 10 days [58]. Persistent restricted diffusion beyond 10 days further differentiates MoCD from HIE.

Cytotoxic edema was not universal and followed a consistent temporospatial pattern with earlier and more severe involvement of parietal and occipital lobes, posterior frontal lobes (pre‐central gyrus, peri‐rolandic area), and posterior temporal lobes, before anterior (entire) frontal lobes were affected. We also observed sparing of the mesial temporal lobes in all affected patients. This is in keeping with our clinical observation that hearing is preserved in severely affected children who invariably show severe visual impairment because of injury to the peritrigonal optic radiation and the occipital cortex. It is notable that we could find no evidence of postnatal diffusion restriction in the cerebellum. These distinctive features differentiate sulfite toxicity from severe HIE.

Cytotoxic edema, accompanied by volume increase, was present at age 6 days (D1) and 10 days (D2). Persisting volume increase is not a feature of severe hypoxic ischemic brain injury and differentiates MoCD from HIE. Restricted diffusion in anterior fronto‐temporal SWM with no restricted diffusion in the overlying swollen cortex was present as early as age 11 days (C1), 6 days (D1), 5 days (E1) and 1 days (K1), with subsequent progression to cortical restricted diffusion or laminar necrosis on follow‐up exams. Anterior fronto‐temporal cortical vasogenic edema accompanied by increased cortical thickness and simultaneous restricted diffusion in the underlying SWM is an additional helpful imaging feature to differentiate the acute stage of MoCD from typical global HIE [22].

Unsurprisingly, cystic transformation in our patients occurred in the same order as cytotoxic edema and progressed rapidly. Transformation of brain tissue to CSF signal intensity filled cysts was preceded by necrotic liquefaction. In children with acute early postnatal onset of severe encephalopathy and seizures, brain liquefaction was seen as early as at age 10 days (K2) but not yet in others at age 10 days (D2) (Figure 4, Panels A^1^–D^1^) or 11 days (C1). Cystic transformation was present at age 18 days (G1) or 40 days (C2) and cysts were seen only posteriorly while the frontal white matter was still in liquefaction after 21 days in Patient D (D3) (Figure 4, Panels A^2^*–D^2^). Postnatal atrophy evolved as early as from age 20 days (E2) or 21 days (D3).

### 4.4 Effects of cPMP Supplementation

Acute sulfite‐related toxicity quickly leads to irreversible cell death, and it is of particular interest to establish whether neuroimaging can help to determine the neurological outcome of patients undergoing disease‐modifying treatment. We have evidence from two patients with MoCD type A that cPMP treatment can reverse vasogenic edema and prevent progression towards cytotoxic edema and cell death. Patient A was ascertained through his family history and was asymptomatic when we found signs of widespread vasogenic edema but no restricted diffusion in the brain MRI at age 3 days (A1). He became lethargic and required tube feeding just prior to starting cPMP treatment at the age of 7 days. The first follow‐up scan showed resolution of edema accompanied by bi‐frontal volume loss (A2, 93 days). Of note, his older affected sibling without cPMP treatment had succumbed to typical MoCD sequelae at the age of 9 months [16]. Patient A had normal motor development and is attending mainstream schooling with support; he, however, had a delay in expressive speech development and has mild cognitive impairment.

Patient B's diagnosis was ascertained when he was screened for metabolic abnormalities due to early postnatal hypoglycaemia. He was irritable and hyperexcitable when his scan revealed signs of vasogenic edema at the age of 4 days, 1 day prior to treatment start (B1). The edema had resolved 9 days after starting cPMP treatment (Figure 5, Panels B–B^1^). We observed a mild degree of brain swelling initially with subsequent mild volume loss but no evidence of atrophy in all subsequent imaging. Mildly increased T2 signal in SWM and DWM persisted until the age of 2 years and was not accompanied by restricted diffusion. This observation reflects impaired or delayed myelination that normalized over time. Patient B has a biochemically severe defect. With ongoing cPMP treatment, he had normal motor development, although his expressive speech is severely delayed, and he has a social communication disorder on the background of mild cognitive impairment [19].

Four patients with MoCD type A were started on treatment with cPMP after the onset of seizures and clinical signs of profound encephalopathy. Patients D, E, G, and I displayed unequivocal signs of encephalopathy and seizures within 48 h after birth and were commenced on cPMP treatment at the age of 7.5, 3.5, 5.0, and 4.25 days, respectively. These 4 patients already showed widespread diffusion restriction in their first brain MRI at ages 6, 5, 18, and 6 days, respectively. All four patients showed a rapid and complete biochemical correction of the metabolic disorder upon treatment with cPMP. Signs of vasogenic edema persisted longer than in Patients A and B, being still present 13, 14, and 16 days after treatment start (G1, D3, E2), but absent after 86 days in one patient (G2). While bi‐frontal WM showed persistent edema, other areas previously affected with cytotoxic edema had progressed to a stage of liquefaction or were already replaced by cysts (D3, E2, I2), indicating a higher vulnerability of occipital and parietal subcortical regions. Of note, signs of vasogenic edema were still absent 23 days after treatment discontinuation in Patient I (I3), reflecting the long biological half‐life of reconstituted sulfite oxidase holoenzyme in vivo and the persistent treatment effect of cPMP [26].

As expected, residual restricted diffusion in the cerebral cortex, thalamo‐capsular regions, and brainstem WM tracts persisted under cPMP treatment when patients were re‐scanned during follow‐up. Diffusion restriction in SWM was replaced with liquefaction of affected tissues and subsequently showed extensive cystic transformation. All four patients developed severe cerebral palsy, severe visual and cognitive impairment, and showed no progression of motor development. Their neurological presentation was identical to untreated patients.

## 5 Conclusions

The universal presence of intrauterine developmental disruption in severe MoCD, even in cases where there was no evidence of prior acute toxic injury, implies that postnatal treatment is not likely to yield a completely normal neurological outcome. This has implications for the assessment of clinical effectiveness of any postnatal intervention.

Acute sulfite‐related toxicity causes a distinctive pattern of brain injury, with evidence of a postero‐anterior gradient of cortical GM and WM vulnerability, early and severe involvement of basal ganglia and subcortical white matter, followed by thalamus and long white matter tracts, but sparing of mesial temporal lobes, and without acute involvement of the cerebellum, as well as prolonged persistence of edema and diffusion restriction. Signs of brain injury can be asymmetric and may indicate sequential insults. This pattern clearly differs from that seen in severe HIE.

Finding disproportionately severe widespread diffusion restriction followed by cortical laminar necrosis, basal ganglia necrosis, and extensive WM cystic transformation in a neonate or young infant without a respective history of perinatal injury can point to sulfite‐related brain injury and should not generally be ascribed to an unwitnessed intrauterine or perinatal hypoxic event.

Diffusion restriction in the context of a sulfite intoxication disorder indicates the presence of cytotoxic edema that will proceed to tissue necrosis and associated neurological sequelae. Decreased consciousness and recurrent seizures in newborns with MoCD are the clinical correlate of widespread cytotoxic edema and are prognostic of a poor functional neurological outcome. Treatment with cPMP leads to rapid biochemical correction in MoCD type A and can prevent disease progression from the reversible stage of vasogenic edema toward irreversible brain injury.

## Author Contributions

**B.C.S.:** investigation, resources, analysis, writing – review and editing. **R.S.:** investigation, analysis, writing – review and editing. **J.A.M.W.:** investigation, analysis, writing – review and editing. **J.P.:** investigation, conceptualization, analysis, writing – review and editing.

## Ethics Statement

This study abided by all applicable laws and regulations, including but not limited to Good Clinical Practices (GCP) and applicable privacy laws. Patient data were de‐identified, and given the limited clinical information provided, ethical review and specific consent for this work were not sought. More detailed clinical data for most patients were previously published, with respective consent.

## Conflicts of Interest

B.C.S. reports an unconditional educational grant and clinical trial sponsorship (paid to institution) for Origin Biosciences Inc. and advisory board participation for Bridge Bio Inc. The other authors declare no conflicts of interest.

## Supporting information


**Figure S1:** Recurrence of vasogenic edema during ongoing treatment with cPMP in Patient G. Upper row: Axial T2WI at the level of centrum semiovale. A: age 18 days, B: age 3 months, C: age 1 year. A: High T2 signal in the frontoparietal subcortical white matter and cortex indicates vasogenic edema, more extensive posteriorly. Deep frontoparietal white matter is relatively spared (white asterisks). B: Interval brain atrophy, parietal ulegyria (blue arrow) and bilateral subdural hemorrhage (orange arrows). Deep frontoparietal white matter shows no vasogenic edema (white asterisks). C: Bilateral high T2 signal in the frontoparietal white matter associated with swelling indicates new vasogenic edema (black asterisks). C1: Single voxel MR Spectroscopy, TE = 144 ms from the right frontoparietal deep white matter demonstrates inverted Lactate doublet (red oval). Note reduced NAA peak (white arrow).

**Table S1:** Clinical characteristics of MoCD patients. All patients were normocephalic at birth and had no history of pregnancy complications or perinatal asphyxia. Onset of clinical symptoms is given with age in days [d]. SV, self‐ventilating.

**Table S2:** Cerebellar dimensions in 13 term newborns with molybdenum cofactor deficiency and corresponding expected gestational age for size [59; 60]. Day of birth is day 0. Transverse cerebellar diameter and vermis height are given in mm. *Inferior vermian hypoplasia present.

**Table S3:** Spatial distribution of cerebral diffusion restriction in brain MRI in 11 patients with molybdenum cofactor deficiency and onset of symptoms on day 1 of life. Patient L already showed signs of established cystic encephalomalacia at age 6 days, prenatal onset of acute encephalopathy was assumed and the scan assigned to disease stage 3. Disease Stage 1: within 7 days of onset; Stage 2: 7–13 days after onset. Stage 3: 14–20 days after onset; Stage 4: 21–27 days after onset; Stage 5: 28 days and more after onset. DWI sequence was not obtained in D3 (21 days).

## Data Availability

The data that support the findings of this study are available on request from the corresponding author. The data are not publicly available due to privacy or ethical restrictions.
