# Supplementary figures and images for: Brain Magnetic Resonance Imaging of Children With Molybdenum Cofactor Deficiency
Source: J Inherit Metab Dis. 2025 Aug 31;48(5):e70079. doi: 10.1002/jimd.70079 (PMC12399460; doi:10.1002/jimd.70079)

## Slide 1
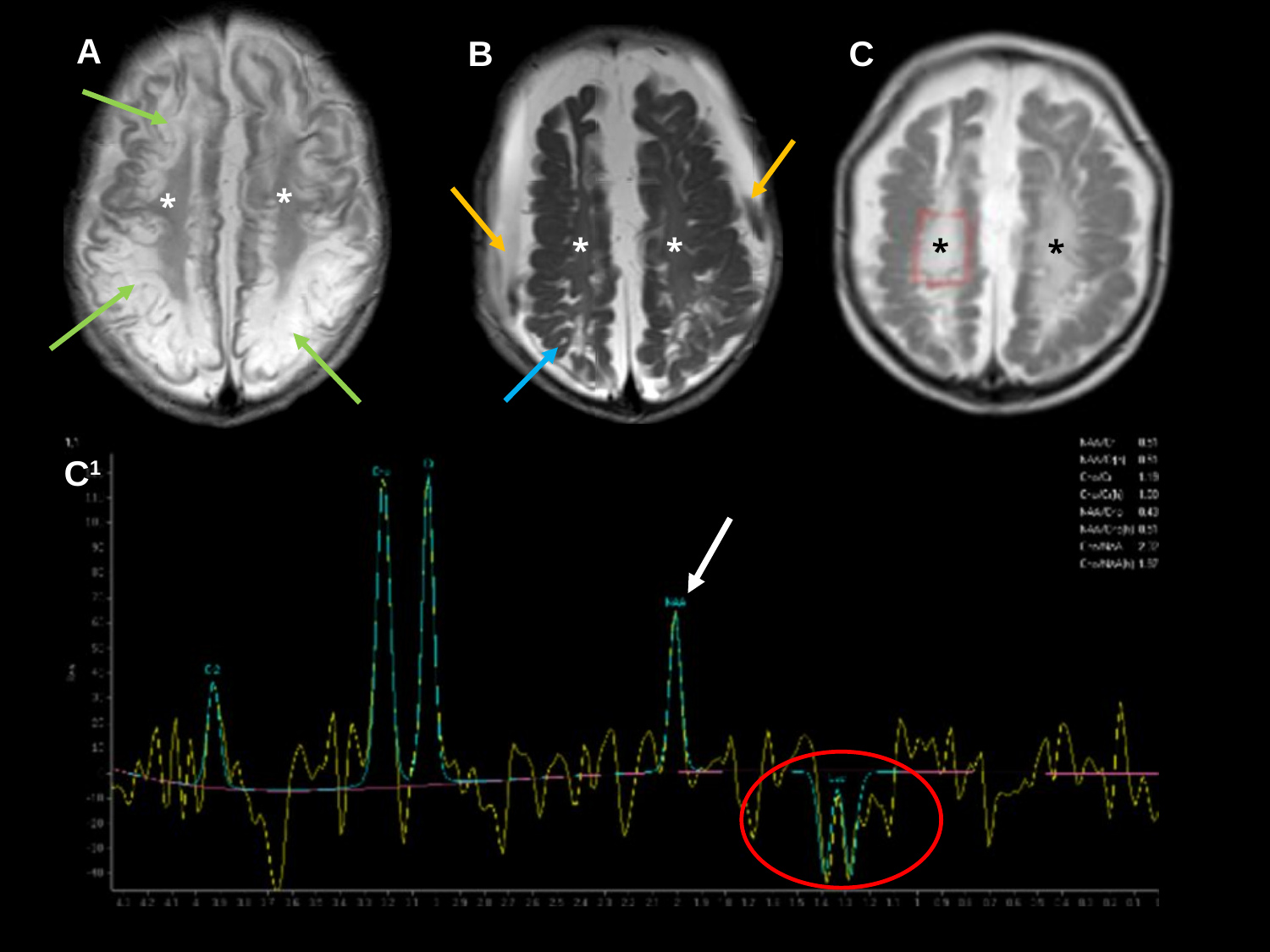

A
C
B
*
*
*
*
*
*
C1

Supplement: Supplementary file 1 — Figure S1: Recurrence of vasogenic edema during ongoing treatment with cPMP in Patient G. Upper row: Axial T2WI at the level of centrum semiovale. A: age 18 days, B: age 3 months, C: age 1 year. A: High T2 signal in the frontoparietal subcortical white matter and cortex indicates vasogenic edema, more extensive posteriorly. Deep frontoparietal white matter is relatively spared (white asterisks). B: Interval brain atrophy, parietal ulegyria (blue arrow) and bilateral subdural hemorrhage (orange arrows). Deep frontoparietal white matter shows no vasogenic edema (white asterisks). C: Bilateral high T2 signal in the frontoparietal white matter associated with swelling indicates new vasogenic edema (black asterisks). C1: Single voxel MR Spectroscopy, TE = 144 ms from the right frontoparietal deep white matter demonstrates inverted Lactate doublet (red oval). Note reduced NAA peak (white arrow). [file JIMD-48-0-s001.pptx]
