# Supplementary material for: Brain Magnetic Resonance Imaging of Children With Molybdenum Cofactor Deficiency
Source: J Inherit Metab Dis. 2025 Aug 31;48(5):e70079. doi: 10.1002/jimd.70079 (PMC12399460; doi:10.1002/jimd.70079)
Supplement: Supplementary file 2 — Table S1: Clinical characteristics of MoCD patients. All patients were normocephalic at birth and had no history of pregnancy complications or perinatal asphyxia. Onset of clinical symptoms is given with age in days [d]. SV, self‐ventilating. [file JIMD-48-0-s003.docx]

**Supplementary Table 3.** Clinical characteristics of MoCD patients. All patients were normocephalic at birth and had no history of pregnancy complications or perinatal asphyxia. Onset of clinical symptoms is given with age in days [d]. SV, self-ventilating.

| Patient ID | MoCD type | First symptom | cPMP started | Short term course | Long-term outcome |
| --- | --- | --- | --- | --- | --- |
| A | A | 5d | 7d | EEG abn 2d, lethargy and abnormal posturing 5d, SV, irritability 6d, full oral feeding from 12d, no seizures | Speech delay, mild learning difficulties, no movement disorder, normocephalic. Alive. |
| B | A | 3d | 5d | Hypoglycemia 0.5d, poor feeding 3d, irritability 4d, SV, no seizures | Speech delay, mild learning difficulties, no movement disorder, normocephalic. Alive. |
| C | B | 0.5d | N/A | Seizures 0.5d, poor feeding, intractable seizures 6d, SV, tube feeding requirement, dystonia | Severe dystonic cerebral palsy, developmental arrest, microcephalic. Died aged 1.2y. |
| D | A | 0.5d | 7.5d | Seizures and apneas 0.5d, coma, ventilated until 3d, intractable seizures, tube feeding required | Severe dystonic cerebral palsy, developmental arrest, microcephalic. Died aged 1.5y. |
| E | A | 1d | 3.5d | Feeding difficulties, first seizure 2d, coma, required ventilation until 12d, intractable seizures | Severe dystonic cerebral palsy, developmental arrest, microcephalic. Died aged 2.8y. |
| F | B | 1d | N/A | Born SGA, irritable, abnormal movements, refractory seizures from 1d, SV with support, tube fed | Severe dystonic cerebral palsy, developmental arrest, microcephaly. Died aged 10m. |
| G | A | 1d | 5d | Irritable 0.5d, refractory seizures 1d, coma, ventilatory support until 6d, tube feeding required | Severe dystonic cerebral palsy, developmental arrest, microcephalic. Alive. |
| H | A | ? | N/A | Irritable and poor feeding 0.5d, possible seizures 1d, recovered, confirmed seizures and first admission 40d | Severe dystonic cerebral palsy, developmental arrest, microcephalic. Died aged 4y. |
| I | A | 0.5d | 4d | Poor feeding 0.5d, irritable and refractory seizures at 1.5d, tube feeding required | Severe dystonic cerebral palsy, developmental arrest, microcephalic. Alive. |
| J | B | ? | N/A | Mild hypotonia 0.5d, oral feeding, abnormal elective cranial US, diagnosis age 1m, later refractory seizures | Severe dystonic cerebral palsy, developmental arrest, microcephalic. Died aged 8m. |
| K | B | 0d | N/A | Seizures from age 4h, encephalopathic 1d, tube feeding required | Cerebral palsy, microcephalic. Died aged 3m. |
| L | B | 0d | N/A | Hypotonia at birth, tube feeding and temporary non-invasive respiratory support. Seizures at 3 weeks. | Severe dystonic cerebral palsy, microcephalic. Died aged 10m. |
| M | B | 0d | N/A | Jittery from birth, increased tone and seizures on day 1, tube feeding required | Severe dystonic cerebral palsy, developmental arrest, microcephalic. Died aged 2.5y. |
