# Supplementary material for: Brain Magnetic Resonance Imaging of Children With Molybdenum Cofactor Deficiency
Source: J Inherit Metab Dis. 2025 Aug 31;48(5):e70079. doi: 10.1002/jimd.70079 (PMC12399460; doi:10.1002/jimd.70079)
Supplement: Supplementary file 3 — Table S2: Cerebellar dimensions in 13 term newborns with molybdenum cofactor deficiency and corresponding expected gestational age for size [59; 60]. Day of birth is day 0. Transverse cerebellar diameter and vermis height are given in mm. *Inferior vermian hypoplasia present. [file JIMD-48-0-s004.docx]

**Supplementary Table 4.** Cerebellar dimensions in 13 term newborns with molybdenum cofactor deficiency and corresponding expected gestational age for size [Hill et al. 1990; Robinson et al. 2007]. Day of birth is day 0. Transverse cerebellar diameter and vermis height are given in mm. *Inferior vermian hypoplasia present.

| **Patient** | **Gestational Age at birth** | **Age at scan** | **Cerebellar hemispheres** | | **Cerebellar vermis** | |
| --- | --- | --- | --- | --- | --- | --- |
| **ID** | **[weeks]** | **[days]** | **Transverse Diameter** | **Equivalent to GA [weeks]** | **Craniocaudal height** | **Equivalent to GA [weeks]** |
| A | 39 | 3 | 47.5 | 35.6 | 23.4 | 39.7 |
| B | 38.5 | 4 | 47.0 | 35.4 | 23.7 | 40.1 |
| C | 39 | 11 | 45.5 | 34.7 | 21.0 | 36.5 |
| D | 39 | 6 | 47.3 | 35.5 | 21.7 | 37.4 |
| E | 40 | 3 | 48.0 | 35.8 | 21.3* | 36.9 |
| F | 39 | 3 | 43.0 | 33.6 | 18.0 | 32.3 |
| G | 41 | 18 | 50.5 | 36.8 | 24.0 | 40.6 |
| H | 40 | 42 | 54.5 | 38.3 | 24.0 | 42.2 |
| I | 39 | 6 | 52.2 | 37.6 | 16.2* | 29.9 |
| J | 40 | 23 | 48.4 | 36.0 | 22.7 | 38.8 |
| K | 39 | 1.5 | 47.2 | 35.5 | 21.5 | 37.1 |
| L | 37.5 | 6 | 46.5 | 35.2 | 19.4 | 34.3 |
| M | 38.3 | 1.5 | 46.0 | 35.0 | 19.0* | 33.7 |
