# Supplementary material for: Brain Magnetic Resonance Imaging of Children With Molybdenum Cofactor Deficiency
Source: J Inherit Metab Dis. 2025 Aug 31;48(5):e70079. doi: 10.1002/jimd.70079 (PMC12399460; doi:10.1002/jimd.70079)
Supplement: Supplementary file 4 — Table S3: Spatial distribution of cerebral diffusion restriction in brain MRI in 11 patients with molybdenum cofactor deficiency and onset of symptoms on day 1 of life. Patient L already showed signs of established cystic encephalomalacia at age 6 days, prenatal onset of acute encephalopathy was assumed and the scan assigned to disease stage 3. Disease Stage 1: within 7 days of onset; Stage 2: 7–13 days after onset. Stage 3: 14–20 days after onset; Stage 4: 21–27 days after onset; Stage 5: 28 days and more after onset. DWI sequence was not obtained in D3 (21 days). [file JIMD-48-0-s002.docx]

**Supplementary Table S5.** Spatial distribution of cerebral diffusion restriction in brain MRI in 11 patients with molybdenum cofactor deficiency and early onset of symptoms. Patient L already showed signs of established cystic encephalomalacia at age 6d, suggesting prenatal onset of acute encephalopathy, and the scan was assigned to disease stage 3. Disease Stage 1: within 7 days of onset; Stage 2: 7-13 days after onset. Stage 3: 14-20 days after onset; Stage 4: 21-27 days after onset; Stage 5: 28 days and more after onset. DWI sequence was not obtained in D3 (21d).

| **Pat ID** | **Age at MRI** | **Disease Stage** | **Anatomical Structures** | | | | | | | | | | |
| --- | --- | --- | --- | --- | --- | --- | --- | --- | --- | --- | --- | --- | --- |
|  |  |  | Cerebral Hemispheres | | | | Deep Grey Structures | | | | Brainstem WM | | |
|  |  |  |  |  |  |  | Basal Ganglia | | | Thalamus |  |  |  |
|  |  |  | Cortical grey matter (CGM)** | Subcortical white matter (SWM)** | Corpus callosum (CC) | Posterior Limb of Internal capsule (PLIC) | Caudate Nucleus (CN) | Putamen (Put) | Globus pallidus (GP) | Thalamus (Thal) | Midbrain (MB) Cerebral peduncles | Pons (PO) Ventral | Medulla oblongata (MO) |
| C1 | 11 | 2 | X^1^ | X | X | X | X | X | X | X^9^ | X | X | X |
| C2 | 40 | 5 | X | X^4^ |  | X^8^ |  |  |  |  |  |  |  |
| D1 | 6 | 1 | X^2^ | X | X | X^8^ | X | X | X | X | X | X | X |
| D2 | 10 | 2 | X | X | X | X | X | X | X | X | X | X | X |
| E1 | 5 | 1 | X^2^ | X | X | X | X | X | X | X^9^ | X | X | X |
| E2 | 20 | 3 | X |  |  | X |  |  |  | X^10^ | X | X | X |
| F1 | 3 | 1 | X | X | X | X | X |  |  | X^9^ | X | X | X |
| G1* | 18 | 3 | X | X | X | X^8^ | X | X | X | X | X | X | X |
| H1 | 42 | 5 | X^3^ | X^6^ |  |  |  |  |  |  |  |  |  |
| I1 | 6 | 1 | X | X | X | X | X | X | X | X^9^ | X | X | X |
| I2 | 24 | 4 | X |  |  | X | X | X | X |  |  | X |  |
| I3 | 48 | 5 | X^4^ |  |  |  |  |  |  |  |  |  |  |
| J1 | 23 | 4 | X |  |  | X |  |  |  |  | X | X |  |
| K1 | 1.5 | 1 | X^1^ | X | X | X^8^ | X | X | X | X^10^ | X | X | X |
| K2 | 10 | 2 | X | X | X | X^8^ | X | X | X | X^10^ | X | X | X |
| K3 | 44 | 5 | X |  |  | X |  |  |  | X^10^ |  |  |  |
| L1 | 6 | 3 | X^5^ | X^5^ | X^7^ |  |  |  |  |  |  |  |  |
| M1* | 1.5 | 1 | X^4^ |  |  |  | X | X | X |  |  |  |  |

*Affected substructures denoted by superscripts*

* Poor quality DWI sequence

** Excluding mesial temporal lobes

1 Excluding anterior frontal and temporal CGM

2 Excluding anterior frontal CGM

3 Right cerebral and left frontal CGM

4 Peri-Rolandic CGM or SWM

5. Unilateral CGM and SWM in posterior temporal, parietal and occipital lobes

6 Right cerebral and left frontal SWM

7 Splenium

8 Internal Capsule

9 Pulvinar

10 Ventro-lateral thalami along PLICs
